# Letrozole and clomiphene versus letrozole alone for ovulation induction in women with PCOS: a systematic review and meta-analysis

**DOI:** 10.61622/rbgo/2025rbgo21

**Published:** 2025-04-30

**Authors:** Karine Eskandar, Juliana Almeida Oliveira, Sandro Augusto Ribeiro, Matheus Pedrotti Chavez, Ana Isabela de Araujo Zotti, Yasmin Jardim Meirelles Dias, Andrea Mora de Marco Novellino

**Affiliations:** 1 Pontificia Universidade Católica do Paraná Department of Medicine Curitiba PR Brazil Department of Medicine, Pontificia Universidade Católica do Paraná, Curitiba, PR, Brazil.; 2 Department of Medicine Faculdade de Minas Belo Horizonte MG Brazil Department of Medicine, Faculdade de Minas, Belo Horizonte, MG, Brazil.; 3 Department of Medicine Faculdade São Leopoldo Mandic Campinas SP Brazil Department of Medicine, Faculdade São Leopoldo Mandic, Campinas, SP, Brazil.; 4 Universidade Federal de Santa Catarina Department of Medicine SC Brazil Department of Medicine, Universidade Federal de Santa Catarina, SC, Brazil.; 5 Faculdades Pequeno Príncipe Department of Medicine Curitiba PR Brazil Department of Medicine, Faculdades Pequeno Príncipe, Curitiba, PR, Brazil.; 6 Washington University. St. Louis Department of Medicine United States Department of Medicine, Washington University. St. Louis, United States.; 7 Universidade Federal do Paraná Department of Toco Gynecology Curitiba PR Brazil Department of Toco Gynecology, Universidade Federal do Paraná, Curitiba, PR, Brazil.

**Keywords:** Clomiphene, Letrozole, Polycystic ovary syndrome, Infertility, female, Ovulation, Ovulation induction

## Abstract

**Objective:**

We aimed to compare the efficacy and safety of letrozole and clomiphene versus letrozole alone for ovulation induction in patients with Polycystic Ovary Syndrome (PCOS).

**Data Sources:**

We systematically searched EMBASE, PubMed, and Cochrane databases on October 31, 2024.

**Study selection:**

We included studies of women with PCOS treated with a combination of clomiphene and letrozole or letrozole alone to induce ovulation that reported any of the outcomes of interest, namely rate of mature follicles and ovulation, ovulation, pregnancy, miscarriages, endometrial thickness, and number of mature follicles.

**Data collection:**

We pooled odds ratios (OR) and mean difference (MD) with 95% confidence intervals (CI) using a random effects model using R statistical software, version 4.2.1. Heterogeneity was assessed with I^2^ statistics, and a random effects model was used.

**Data Synthesis:**

Four RCTs and two observational studies comprising 592 patients were included. Combined therapy was associated with a higher rate of a mature follicle (OR 2.74; 95% CI 1.72-4.37; p< 0.001; I^2^=0%) and ovulation (OR 2.55; 95% CI 1.57-4.12; p< 0.001; I^2^=35.9%). The number of mature follicles, number of pregnancies, thickness of endometrial lining, and the incidence of adverse events, including headache, abdominal bloating, fatigue, back pain, breast discomfort, and night sweats, were similar between groups.

**Conclusion:**

In women with anovulatory infertility secondary to PCOS, letrozole and clomiphene citrate combined therapy was associated with improved mature follicle and ovulation rates, with a similar safety profile compared to letrozole alone. However, no significant impact was observed on pregnancy rates.

## Introduction

Polycystic ovary syndrome (PCOS) is the most common cause of anovulatory infertility, affecting 5–25% of women of reproductive age worldwide.^(1-4)^ Clomiphene citrate (CC) has been largely prescribed for PCOS over the past 50 years. It inhibits the negative feedback effects of estrogen at hypothalamic and pituitary levels, increasing follicle-stimulating hormone (FSH) levels and stimulating follicular maturation.^(5)^ Although clomiphene achieves an ovulation rate of 80%, it also diminishes the favorable effects of estrogen on the endometrial lining and cervical mucus, leading to a pregnancy rate of only 40%, approximately.^(6-9)^

Another therapeutic option is letrozole, an aromatase inhibitor that decreases estrogen biosynthesis and central negative feedback, which increases FSH levels without adversely affecting the endometrial lining.^(10,11)^ In 2018, guidelines established by the European Society of Human Reproduction and Embryology recommended letrozole as the preferred initial therapy for ovulation induction in women with PCOS.^(12)^ This recommendation was based on its superior efficacy in achieving pregnancy and live birth rates when compared with CC.^(10)^

Due to their differing mechanisms of action, several studies evaluated the combination of letrozole and CC in increasing fertility outcomes. However, uncertainty remains about the efficacy and safety of this combination. In this study, we conducted the first systematic review and meta-analysis assessing the efficacy and safety of letrozole and CC compared with letrozole alone on ovulation in women diagnosed with PCOS.

## Methods

This systematic review and meta-analysis was prospectively registered in the International Prospective Register of Systematic Reviews (PROSPERO), under registration number CRD42023465036 and performed following the Cochrane Collaboration Handbook for Systematic Review of Interventions and the Preferred Reporting Items for Systematic Reviews and Meta-Analysis (PRISMA) statement guidelines.^(13,14)^

### Eligibility criteria and study selection

We included studies that satisfied the following eligibility criteria: (1) randomized controlled trials (RCTs), cohort, or case-control studies; (2) among patients diagnosed with anovulatory infertility secondary to PCOS; (3) comparing the combination of letrozole and clomiphene versus letrozole alone; and (4) reporting any of the outcomes of interest. We excluded studies with (1) other causes of infertility; (2) anovulatory infertility of uncertain causes; (3) no outcomes of interest; or (4) overlapping patient populations. There were no language restrictions.

### Search strategy and data extraction

We systematically searched MEDLINE, Embase, and Cochrane Library databases from inception to October 31, 2024. Our search strategy used a combination of the following keywords: "aromatase inhibitors", letrozole, Femara, Anastrozole, Arimidex, Exemestane, Aromasin, clomiphene, PCOS, "polycystic ovarian syndrome", and "Polycystic Ovary Syndrome". All records retrieved were independently assessed by 2 reviewers and all disagreements were resolved by a third reviewer. Full texts were reviewed and discussed by four authors for evaluation of eligibility criteria. Conference abstracts and prospective trial registries were also searched. In addition, the references from all included studies, previous systematic reviews, and meta-analyses were searched manually for any additional studies. Two authors independently extracted data in spreadsheets following predefined search criteria. We extracted data for (1) women with follicles >15mm; (2) ovulation; (3) number of follicles >15mm; (4) pregnancy; (5) pregnancy loss; (6) endometrial thickness; (7) night sweats; (8) headache; (9) fatigue; (10) bloating; and (11) back pain.

### Risk of bias

The Cochrane Collaboration's tool for assessing the risk of bias in randomized trials (Rob 2) was used for the assessment of each RCT.^(15)^ Non-randomized studies were evaluated utilizing the risk of bias in non-randomized studies of interventions (ROBINS-I).^(16)^ Two independent authors conducted the risk of bias assessment (K.E. and A.I.A.Z.). Disagreements were resolved by consensus. Publication bias was evaluated through visual inspection of funnel-plot graphs to check for symmetrical distribution of trials with similar weights.

### Statistical synthesis

We pooled continuous outcomes using mean differences (MD) and binary endpoints using odds ratios (OR) with 95% confidence intervals (CI). For continuous variables reported other than (mean ± standard deviation), conversions were adopted, and skewness detection was also conducted.^(17-19)^ We assessed heterogeneity with the Cochrane Q-test and I^2^ statistics; P values ≤ 0.10 and I^2^ values >25% were considered significant for heterogeneity. Furthermore, I^2^ cutoffs for low, moderate, and high risk of heterogeneity were <25%, 26-50%, and >50%, respectively.^(20)^ Statistical significance was defined as a p-value <0.05. We used random-effects models for all outcomes regardless of their heterogeneity. Statistical analyses were performed using R statistical software, version 4.2.1 (R Foundation for Statistical Computing), and its "meta" package.

## Results

### Study selection and characteristics

**Figure 1:**
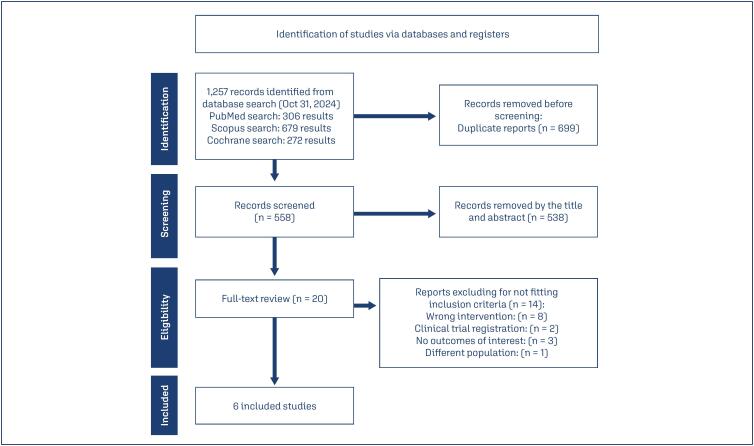
PRISMA flow diagram of study screening and selection

As detailed in figure 1, the initial search yielded 1,257 results. After removing duplicate records and articles that did not meet inclusion criteria based on title or abstract analysis, 20 studies were retrieved for a comprehensive review. Finally, six studies involving 592 women were included. Four were RCTs,^(21-24)^ one was a retrospective cohort study,^(25)^ and another was a prospective cohort.^(26)^ The mean age was 28.47 ± 4.86, average BMI was 28.18 ± 5.44, and oligomenorrhea was observed in 61.8% of patients. Baseline study characteristics are detailed in chart 1.

**Chart 1:** Baseline characteristics of included studies

Study	Design	Patients, n	Age, y	BMI kg/m^2^	Duration of infertility, y	Oligomenorrhea, n	HA^¥^, n
Mejia et al. (2019)^(21)^	RCT	35	31±3.9	33±8.7	30 ± 29.6*	34	20
		35	30±4.4	34±7.0	28 ± 18.5*	33	21
Mejia et al. (2024)^(22)^	RCT	92	-	-	-	-	-
		98	-	-	-	-	-
Mirajkar et al. (2023^)(26^)	Prospective Cohort	32	29.02 ± 7.9	28.1 ± 3.7	4.98 ± 4.0	56	32**
		38					
Panda et al. (2023)^(23)^	RCT	40	28.1 ± 3.2	28.6 ± 3.5	3.65 ± 1.67	-	-
		40	27.6 ± 3.3	29.09 ± 3.4	4.12 ± 1.6	-	-
Sharma et al. (2023)^(25)^	Retrospective Cohort	40	28.05 ± 4.45	25.21 ± 3.28	5.33 ± 3.08	34	11
		40	28.13 ± 3.68	25.16 ± 2.76	4.25 ± 2.03	33	14
Zamaniyan et al. (2023)^(24)^	RCT	51	28.74±3.8	26.58 ± 4.4	2.83 ± 1.8	4	12
		51	28.75± 4.2	26.23 ± 4.5	3.95 ± 2.2	5	18

* Mean ± SD in months;

** number of patients with hirsutism with non-specified criteria;

¥ HA: number of patients with clinical or laboratorial hyperandrogenism; LECC: Letrozole and clomiphene citrate; LE: Letrozole alone

Diagnosis of PCOS was based on the Rotterdam criteria.^(27)^ Ovulation was identified when serum progesterone level was ≥ 3-5 ng/mL. Pregnancy was identified based on increased serum or urine β-human chorionic gonadotropin or by sonographic evidence of an intrauterine gestational sac. Follicle number and size along with endometrial thickness were determined by transabdominal or transvaginal ultrasound. Interventions across all studies included the administration of a daily dose of letrozole with doses ranging from 2.5 mg to 7.5 mg and CC with doses ranging from 50 mg to 100 mg on days 3 through 7 of the menstrual cycle. The control arm consisted of daily doses of letrozole alone, ranging from 2.5 mg to 7.5 mg daily. In two studies, patients also received an injection of human chorionic gonadotropin (hCG) of 5000 IU when at least one of the leading follicles attained a diameter of 18 mm.^(23,24)^ Outcomes other than ovulation and pregnancy were measured before this intervention. Other key design features of the included studies are detailed in chart 2.

**Chart 2:** Key design features in included trials

Study	Mejia et al. (2019)^(21)^	Mejia et al. (2024)^(22)^	Mirajkar et al. (2023)^(26)^	Panda et al. (2023)^(23)^	Sharma et al. (2023)^(25)^	Zamaniyan et al. (2023^)(24^)
Country	United States	United States	India	India	India	Iran
Design	RCT	RCT	Prospective Cohort	RCT	Retrospective Cohort	RCT
Enrollment period	09/2016 – 03/2018	01/2022 – 12/2023	-	10/2020 – 05/2021	01/2020 – 12/2022	04/2019 – 03/2020
Trial number	NCT02802865	NCT05206448	-	CTRI/2020/09/028012	-	IRCT20160815029374N6
Participants	70	190	70	80	80	102
Treatments	LE 2.5 mg + CC 50 mg daily on cycle days 3-7	LE 2.5 mg + CC 50 mg daily on cycle days 3-7	LE 5 mg + CC 100 mg daily for 5 days	LE 5 mg + CC 100 mg daily for 5 days	LE 2.5 mg + CC 50 mg daily on cycle days 3-7	LE 5 mg + CC 100 mg daily on cycle days 3-7
	LE 2.5 mg daily on cycle days 3-7	LE 2.5 mg daily on cycle days 3-7	LE 5 mg daily for 5 days	LE 5 mg daily for 5 days	LE 2.5 mg daily on cycle days 3-7	LE 5 mg daily on cycle days 3-7
Additional therapy	N/A	Both groups received increments of LE 2.5 mg up to 7.5 mg if anovulatory or no follicular development	N/A	5,000U of hCG if visualized a follicle ≥ 18mm	N/A	5,000U of hCG if visualized a follicle ≥ 18mm
Outcomes	AE, ET, NoF15, PG, M/C, OV, BP, BD, NS, Ft, Bl, HA	PG, OV	ET, NoF15, PG, M/C	ET, NoF15, PG, OV	AE, OV, BP, BD, NS, Ft, Bl, HA	ET, NoF15*, PG, M/C

LE: Letrozole; CC: Clomiphene citrate; AE: adverse events; ET: endometrial thickness; NoF15: Number of follicles ≥15 mm;

* Number of follicles ≥14 mm;

PG: pregnancy; M/C; Miscarriage; OV: ovulation; BP: back pain; BD: breast discomfort; NS: night sweat; Ft: fatigue; HA: headache

### Quality assessment

**Figure 2:**
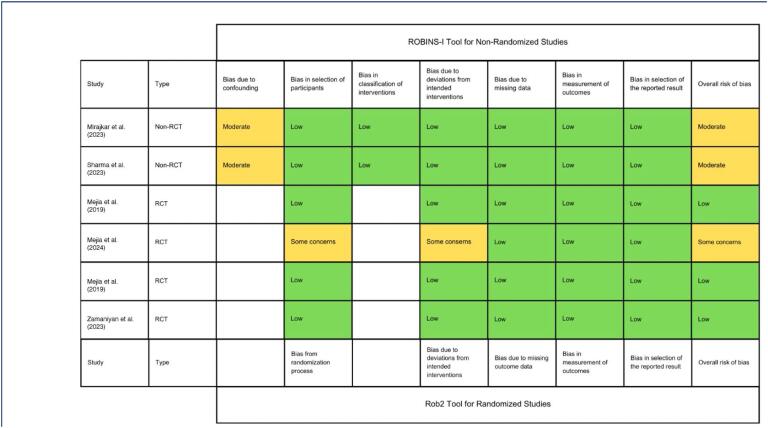
Risk of Bias Assessment

**Figure 3:**
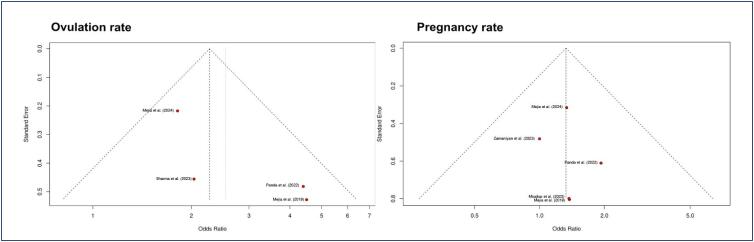
Funnel plots

Three RCTs were deemed at low risk of bias,^(21,23,24)^ while one presented some concerns due to insufficient data in the allocation and deviation from intended intervention domains.^(22)^ Both observational studies had a moderate risk of bias in the confounding domain, as shown in figure 2. Funnel plot analyses of two outcomes showed a symmetrical distribution of studies toward the pooled treatment effect, demonstrating similar weights of studies and no evidence of publication bias (Figure 3).

### Efficacy outcomes

**Figure 4:**
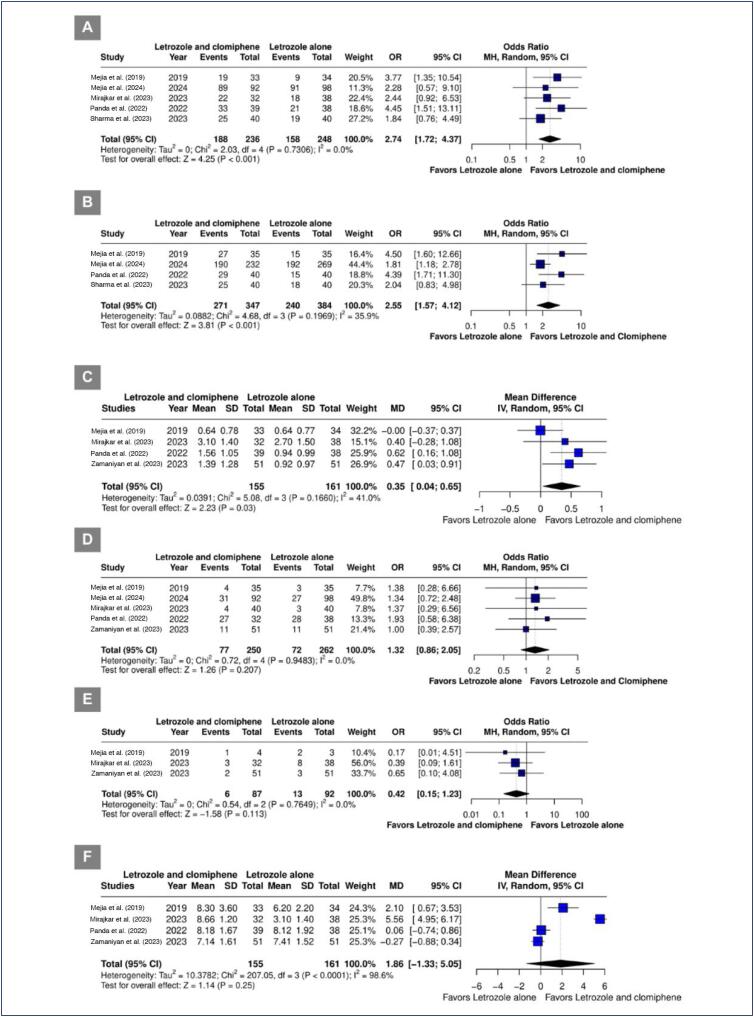
A. Mature follicule rate. B. Ovulation rate. C. Number of mature follicles. D. Pregnancy rate. E. Miscarriages rate. F. Endometrial lining thickness.

Mature follicle rate (OR 2.74; 95% CI 1.72-4.37; p< 0.001; I^2^=0%) (Figure 4A), ovulation rate (OR 2.55; 95% CI 1.57-4.12; p< 0.001; I^2^=35.9%) (Figure 4B), and the number of mature follicles (MD 0.35; 95% CI 0.04 to 0.65; p=0.03; I^2^=41.0%) (Figure 4C) were significantly higher in the combined therapy group. Pregnancy rate (OR 1.32; 95% CI 0.86-2.05; p=0.207; I^2^=0%; Figure 4D), miscarriages rate (OR 0.42; 95% CI 0.15-1.23; p=0.113; I^2^=0%) (Figure 4E), and endometrial lining thickness (MD 1.86 mm; 95% CI -1.33 to 5.05 mm; I^2^=98.6%) (Figure 4F) were similar between groups.

### Safety outcomes

**Figure 5:**
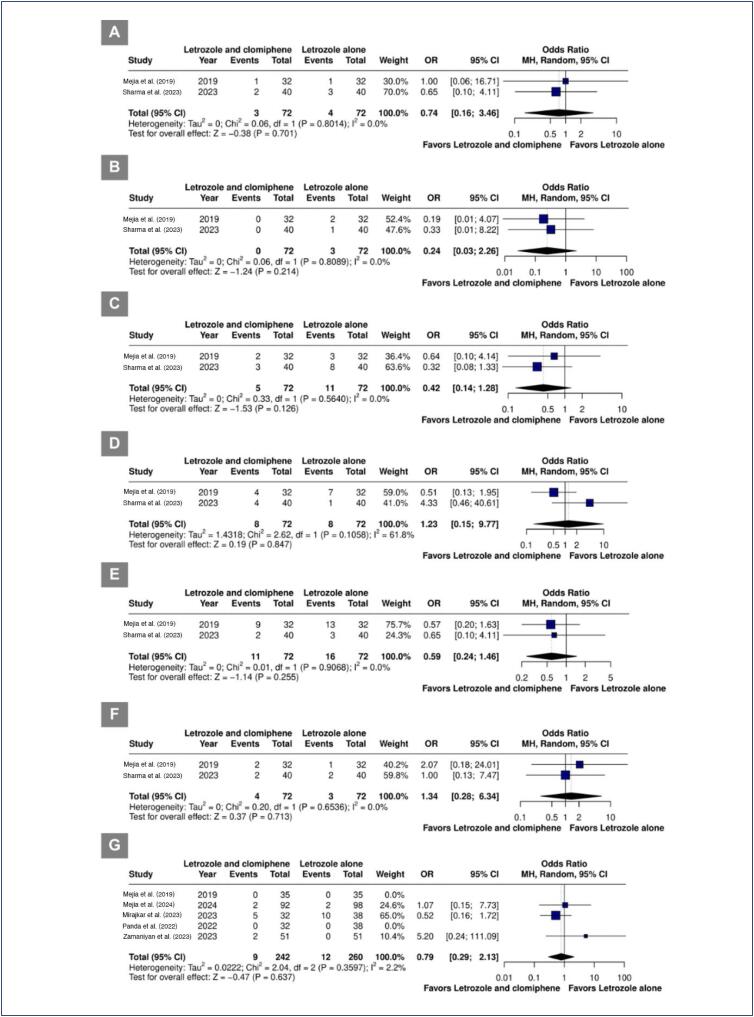
Adverse events. A. Back pain. B. Bloating. C. Breast discomfort. D. Fatigue. E. Headache. F. Night sweat. G. Twin pregnancy rate.

The incidence of adverse events in patients who received combination therapy was not significantly different in comparison with letrozole alone, including back pain, breast discomfort, night sweats, fatigue, bloating, and headache (Figures 5A-F). A post hoc analysis of the twin pregnancy rate also showed similarities between groups (Figure 5G).

## Discussion

In this systematic review and meta-analysis, we assessed the efficacy and safety of letrozole, and CC combined therapy compared with letrozole alone in inducing ovulation in patients with anovulatory infertility secondary to PCOS. The primary findings demonstrated that combined therapy was associated with significantly higher mature follicles and ovulation rates than letrozole alone. We also observed a similar frequency of pregnancy and twin pregnancy despite a higher number of mature follicles in the combined therapy group.

Clomiphene citrate has been used for decades as a first-line treatment for ovulation induction and has been gradually replaced by letrozole since 2018.^(28-30)^ This recommendation is reinforced by a meta-analysis of 29 RCTs comparing letrozole with CC where letrozole therapy was associated with an overall higher rate of ovulation (67.77% vs 58.54%), clinical pregnancies (34.56% vs 23.42%), and live births (32.82% vs 22.17%).^(31)^ While letrozole seems superior to CC, its extended use – up to 10 days per cycle – or stair-step approach with dose increments up to 7.5mg daily does not seem to significantly increase the success rates of ovulation, clinical pregnancies, or live births.^(32,33)^

Letrozole has previously demonstrated a protective role in the endometrial lining, improving endometrial receptivity while maintaining its thickness during ovulation induction.^(34-36)^ Additionally, letrozole is expected to contribute to a superior quality of cervical mucus.^(9)^

The positive association found between increased ovulation and the administration of combined therapy in a single menstrual cycle is supported by an RCT involving 82 women that found an ovulation rate of 80% and a pregnancy rate of 46% when administering combined therapy to patients with anovulatory infertility for up to 4 cycles.^(37)^ Two forthcoming RCTs involving a total of 184 patients plus the complete results of the trial NCT05206448 are expected to provide additional insights, especially regarding clinical pregnancy outcomes.^(38,39)^ The current lack of a significant difference in this outcome is likely attributed to the limited number of participants. The anticipated larger sample size in the upcoming trial is expected to enhance statistical power, enabling more robust conclusions to be drawn.

This study has some limitations. First, two of the studies associated the use of hCG upon the identification of mature follicles.^(23,24)^ This practice is known to increase ovulation rates by enhancing FSH release, which may introduce bias to our findings. Second, there were slight differences among studies in the progesterone levels used to define ovulation, which might have negatively influenced the ovulation outcomes in the study with a higher cutoff of progesterone levels.^(25)^ Third, the number of mature follicles present in this meta-analysis was similar between groups, which may not be accurate due to positively skewed data in one study.^(21)^ This study data had an asymmetric distribution and the conversion of median and interquartile range to mean and standard deviation might have brought some bias to this analysis.^(18,19)^ Lastly, there were some differences in the patient populations across the included studies. For instance, one study focused solely on women resistant to CC.^(26)^ However, this population exhibited a similar therapeutic response when compared with the other studies.

Our meta-analysis supports the use of combined therapy due to its significant benefit in follicle maturation and ovulation without adding new safety concerns.

## Conclusion

This systematic review and meta-analysis support the efficacy and safety of letrozole and clomiphene citrate combined therapy for women with anovulatory infertility secondary to PCOS. This combination promoted a significant benefit in the maturation of follicles and ovulation, with a similar safety profile to letrozole alone. However, no significant impact was observed on pregnancy rates.
